# Mitral Valve Segmentation Using Robust Nonnegative Matrix Factorization

**DOI:** 10.3390/jimaging7100213

**Published:** 2021-10-16

**Authors:** Hannah Dröge, Baichuan Yuan, Rafael Llerena, Jesse T. Yen, Michael Moeller, Andrea L. Bertozzi

**Affiliations:** 1 Department of Electrical Engineering and Computer Science, University of Siegen, 57076 Siegen, Germany; michael.moeller@uni-siegen.de; 2 Department of Mathematics, University of California, Los Angeles, CA 90095, USA; ybcmath@gmail.com (B.Y.); bertozzi@math.ucla.edu (A.L.B.); 3 Non-Invasive Cardiology Department, Keck Medical Center of University of Southern California, Los Angeles, CA 90033, USA; rafael.llerena@med.usc.edu; 4 Department of Biomedical Engineering, University of Southern California, Los Angeles, CA 90089, USA; jesse.yen@usc.edu

**Keywords:** mitral valve, segmentation, robust non-negative matrix factorization, echocardiography

## Abstract

Analyzing and understanding the movement of the mitral valve is of vital importance in cardiology, as the treatment and prevention of several serious heart diseases depend on it. Unfortunately, large amounts of noise as well as a highly varying image quality make the automatic tracking and segmentation of the mitral valve in two-dimensional echocardiographic videos challenging. In this paper, we present a fully automatic and unsupervised method for segmentation of the mitral valve in two-dimensional echocardiographic videos, independently of the echocardiographic view. We propose a bias-free variant of the robust non-negative matrix factorization (RNMF) along with a window-based localization approach, that is able to identify the mitral valve in several challenging situations. We improve the average f1-score on our dataset of 10 echocardiographic videos by 0.18 to a f1-score of 0.56.

## 1. Introduction

The precise movement of the mitral valve is of crucial importance for a proper blood flow [1]. Therefore, accurate detection of mitral valve disease is of great importance for the treatment and prevention of several diseases. Especially in old age, a defect of a heart valve is a common disease. According to Mohty et al. [2], more than 12.5% of the elderly people of 75 years or over suffer from heart valve diseases. In a study in 2006, mitral regurgitation, which is a disease of the mitral valve that does not close tightly and thus partially allows the blood to flow back, was the most measured common kind of heart disease [3].

Very helpful for the recognition of the degree of mitral regurgitation and thus, the necessity of surgical intervention, is the diagnostic imaging procedure of echocardiography. Echocardiography uses ultrasound techniques to receive an image of the heart and has multiple qualities, such as the real-time displaying of heart motion as well as of the blood flow. It is the most common medical imaging technique despite other technologies, such as magnetic resonance and computer tomography [4,5] and benefits, compared to other diagnostic methods, from the portability of its required equipment, its easy implementation and the low price as well as the safety of this type of examination. Additionally, for the affected patient, the ultrasound examination is associated with minimal discomfort and stress [6,7].

The analysis of the echocardiographically recorded data is performed manually by trained specialists, which is slow and subjective and depends on the current stay of the specialist [8,9]. An automated, robust and repeatable method for mitral valve detection would be beneficial in this regard, and the recording and analysis of cardiac ultrasound data by non-experts is of highest interest.

Many applications benefit from the successful automatic segmentation of the mitral valve of the heart in echocardiographic ultrasound videos. For example, in cardiac surgery, the automatic detection of the valve prior to or during surgery could be used as support or for robotic assistance [7]. Another field of application is diagnostics, where segmentation can lead to a better understanding of the behavior and geometry of the patient’s mitral valve and thus of its various diseases [10]. Finally, the segmentation of the mitral valve can support the training of ultrasound diagnosticians [11].

Automatic and unsupervised segmentation of the mitral valve in echocardiographic ultrasound videos was first approached by Zhou et al. [10] by capturing the irregular motion of the mitral valve, using a matrix factorization method. This method was later taken up by Dukler et al. [9], who used robust non-negative matrix factorization (RNMF) for mitral valve segmentation, with the underlying basic concept of distinguishing the rigid motion of the myocardial muscle from the nonrigid and irregular motion performed by the heart valve and thus, being able to segment the latter. Consistent with this work, our method uses a bias-free version of RNMF to capture irregular motion of the mitral valve, followed by an additional variational segmentation technique to perform successful segmentation. Our paper’s contributions are listed below:

1. We use Bregman iteration to avoid the inherent bias of $\ell^{1}$ regularization in the RNMF model within the context of mitral valve segmentation.
2. As an additional variational segmentation technique, we use the Chan–Vese segmentation algorithm [12] to which we add a new regularization term so that it matches the problem of mitral valve segmentation.
3. We proposed a new segmentation refinement algorithm that takes into account the opening and closing motion of the heart valve and, in combination with the unbiased RNMF model and the regularized variational segmentation technique, allows us to perform fully automatic segmentation without any further knowledge about the heart valve.

Our paper is divided into six sections. Section 2 discusses related work dealing with general segmentation and previous semi-automatic and automatic mitral valve segmentation methods. Section 3 discusses our proposed mitral valve segmentation method and is divided into four subsections. The first three subsections cover the crucial steps for fully automatic valve segmentation, consisting of myocardial muscle detection, valve segmentation, and a refinement step. The fourth subsection deals with the optional step of additional myocardial valve detection, using a windowing method. In Section 4, we present the datasets we use to evaluate our method. Section 5 deals with the results of our experiments, which include the analysis of various hyperparameters, comparison with two related methods for cardiac valve segmentation, and discussion of error cases. In the conclusion in Section 6, we provide a brief summary of our results and present limitations for real-world applications as well as possible future novel approaches to improve mitral valve segmentation.

## 2. Related Work

Image segmentation is a commonly used technique with a wide range of applications, including medical imaging. The current state of the art in this field is largely dominated by deep learning approaches, which have also been successful in medical images; see [13]. However, the training of such neural networks usually requires large amounts of training data, which in medical applications, such as echocardiographic videos, are difficult to access in a sufficient amount together with their results.

Moreover, a very difficult to analyze and possibly hidden bias of a deep neural network toward the training data remains a risk in safety critical areas, such as medical image segmentation or disease classification.

While model-based (unsupervised) methods, such as the proposed approach, also contain a bias, this bias arises from the model itself (e.g., from an assumption of temporal or spatial coherence) such that the type of bias and uncertainty of the solution is known a priori.

So, instead of exploiting training data, classical techniques rely on cleverly designed methods, such as thresholding methods, clustering methods, region growing methods and more. An overview of some of these methods is given in [14,15]. More recent works consider variational methods (see, for example, [12,16]), or formulate similar energy minimization problems on suitable graphs, e.g., motivated by Markov random fields or conditional random fields; see [17,18,19] for examples.

The image formation of echocardiographic images differs from photographic images and thus, poses a special challenge in the field of segmentation. Cardiac ultrasound images are generated by measuring the time and amplitude of ultrasound waves sent in the direction of the heart and reflected back from its structures, whereby the intensities of the reflected echoes within an ultrasound image are converted into gray tones. When recording ultrasound videos, the frame rate, or temporal resolution, is determined by the desired field of view, line spacing, and speed of sound in the medium [20]. So, opposed to the segmentation of photographic images, the automatic segmentation of the mitral valve poses various additional challenges, as the mitral valve does not differ from the rest of the heart muscle in structure or intensity, and ultrasound videos often contain strong noise. In addition, the mitral valve does not have a smooth structure or hard edges in its ultrasound recordings; it may occur that in several frames, the mitral valve is hardly visible or not visible at all. Its form and structure also differ according to the considered echocardiographic view.

Such challenges are one of the reasons why many recent techniques have focused on semi-automatic methods that still rely on certain user inputs. Algorithmically, many of these methods use the active contours model of Kass et al. [16], for example, in [8,21], or variants thereof; see [22,23]. Other approaches, e.g., that of Shang et al. [24], exploit level set segmentation methods based on the intensity difference between the mitral valve and the background. The advantage of those semi-automatic methods over manual methods is that often, only one or few user inputs are required, such as drawing the initial contours around the mitral valve, and are, therefore, less time consuming than a manual segmentation of the mitral valve by specialists. However, specialists are still required to draw the initial contour. Important factors for the clinical application of those methods are their reliability and time consumption. According to [21], the method of Kass et al. [16] needs 20 min per ultrasound video, so the method can only be used in applications where no real-time application is required. Due to the speed of their real-time method, Martin et al. [21] suggests them for preoperative and intraoperative scenarios. In general, however, semi-automated and also automated heart valve recognition procedures should be critically reviewed for failure. In particular, [22,23] show that the simultaneous speed and reliability in a method is a great challenge. In [23], a more reliable method is proposed than in [22], but at the expense of speed.

The first automatic, unsupervised method for tracking and segmenting the mitral valve was proposed in 2012 by Zhou et al. [10] and takes advantage of the fast motion of the mitral valve and thus the spectral distribution of the ultrasound video that is strongest in the valve area. Inspired by this, a more robust method, dealing with the low-rank approximation of echocardiographic videos, was proposed by Liu et al. [7], called constrained outlier pursuit, to prevent possibly occurring drifts in tracking the mitral valve. In addition, they use region scalable active contours for segmenting the mitral valve. Based on the method in [10,25], Dukler et al. [9] developed a new method to detect the mitral valve by applying RNMF [26] on multiple areas of the echocardiographic video and measuring the residue at each position.

RNMF is a regularized version of nonnegative matrix factorization (NMF) on which, in 1999, Lee and Sung [27] conducted a study that attracted wide interest in several application areas, including face recognition [28], medical applications [29,30] and general clustering approaches [31]. Various minimization algorithms were presented in the following years to optimize NMF, such as multiplicative updating rules [32] or hierarchical alternating least squares [33] for faster convergence. For an overview of the optimizations, we refer here to [34,35]. To improve the decomposition results of NMF, among others, orthogonality condition [36] or sparsity conditions [26,37,38] were added to NMF; in this paper, we consider the sparsity constraint of [26], known as RNMF, which is a special case of a method used by Corinzia et al. [39], who used neural network matrix factorization combined with a threshold network, which is trained in an unsupervised manner, on each echocardiographic video individually to capture the motion of the mitral valve. In order to define the region of the heart valve, they additionally localized the heart valve region, using optical flow calculated on the outcome of the neural network. In a subsequent paper on this topic, Corinzia et al. [40] extend this work and propose a generalised form of matrix factorisation together with a parameterised threshold operator. To find the region of the mitral valve, they use optical flow and the low-dimensional time series representation of the echocardiographic video. Also, they present post-processing algorithms for improvement of the segmentation.

In line with these prior works that successfully exploited RNMF to detect the position of the mitral valve in the echocardiographic video, we extend [9,40] in the following two ways: (1) We avoid the inherent bias of $\ell^{1}$ regularization in the RNMF model by exploiting Bregman iterations [41], and (2) we utilize an additional variational segmentation technique (a modified version of the Chan–Vese algorithm [12]) on the result of the RNMF instead of relying on plain thresholding. We demonstrate in several numerical experiments that the proposed modifications result in higher quality results specifically on challenging examples, where the mitral valve is moving quickly and thus is difficult to detect. In contrast to [9,40], the segmentation technique allows us to run our algorithm, even without prior windowing techniques (which rely on the manually specified window size that contains the mitral valve) and therefore, represents advances toward fully-automatic mitral valve segmentation.

Table 1 shows a summary of our and previous mitral valve segmentation and tracking methods in echocardiographic recordings, sorts them into subgroups, and provides a listing of their respective advantages and disadvantages.

Related to mitral valve segmentation on echocariographic images is the segmentation of the left ventricle, that is adjacent to the valve. In this area, there have been various approaches in recent years, including energy minimization methods as the method of Pedrosa et al. [42], who combine a shape-based deformable model with a statistical shape model, allowing the joint use of image information as well as shape-based cues for segmentation. A more general approach, which can be applied to ventricle segmentation, was presented by Ciecholewski in 2016 [43], presenting an edge-based active contour model that applies an inflation/deflation force for each node of an active contour, allowing it to shift active contour nodes and locate object boundaries, even in a noisy image while still approximating the rigor of the object shape. In addition, approaches with deep learning elements were studied, such as the method of Carneiro et al. [44], addressing the segmentation of the left ventricle by rigid and non-rigid decoupled segmentation using artificial neural networks, and Ali et al. [45] presenting a deep learning framework that combines ResNet and U-Net elements. Especially useful for comparison is the evaluation framework of Bernard et al. [46] for the comparison and evaluation of left ventricle segmentation algorithms.

## 3. Method

**Figure 1:**
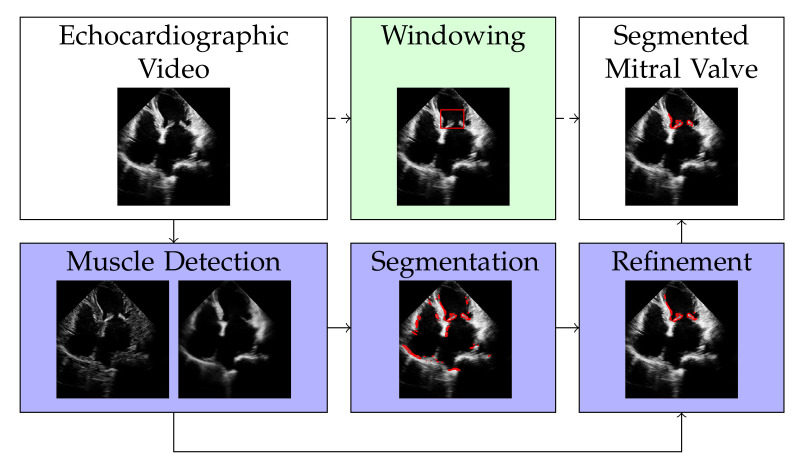
Overview of the proposed method. The white filling of the box shows the input and output of our method. The dark purple fill shows the mandatory steps, while the light green fill indicates the optional step of our algorithm.

The discrete representation of an echocardiographic video with *t* frames is a three-dimensional tensor $X_{3D}\in R_{+}^{s_{x}\times s_{y}\times t}$. By vectorizing the two spatial dimensions $s_{x}$ and $s_{y}$, the video $X_{3D}$ can be represented by a matrix $X\in R_{+}^{s\times t}$ with $s=s_{x}s_{y}$, i.e., with each column of *X* representing a frame of the echocardiographic video. Our approach for segmenting the mitral valve in an echocardiographic video is to first apply RNMF to the matrix *X* to separate the structure of the mitral valve from the structure of the heart muscle. It follows a segmentation and a refinement step, as shown in Figure 1, to enclose the mitral valve structure at its edges. Windowing is an optional step that defines the area where the mitral valve can occur, thus increasing the accuracy of segmentation. However, this (optional) step requires knowledge of the approximate size of the mitral valve. In the following subsections, we will describe each of the steps illustrated in Figure 1 separately.

### 3.1. Muscle Detection

Similar to the classical RNMF, we model the video *X* to be a sum of the three following components:

(1) X=WH+S+N,

where WH represents a low-rank part that captures the static background as well as simple rigid movements, *S* represents the non-rigidly moving parts (i.e., mainly the mitral valve), and *N* is the observation noise. The low-rank constraint is enforced by WH being a product of two matrices $W\in R^{s\times r}$ and $H\in R^{r\times t}$, where *r* is significantly smaller than *s* and *t*. Moreover, assuming *S* to occupy a comparably small spacial area but contain large values gives rise to penalizing the $\ell^{1}$ norm of *S* (in the sense of the sum over the absolute values of *S*), and assuming *N* to be roughly Gaussian distributed gives rise to penalizing the squared $\ell^{2}$ norm of *N*. Combining these modeling assumptions with (physically meaningful) non-negativity constraints on all entries of *W*, *H*, and *S* gives rise to the usual RNMF model as follows:

(2) $$\underset{W,H,S\geq 0,N}{arg min}{∥N∥}_{2}^{2}+{\lambda ||S||}_{1},\;\;s.t.\;(1)\;\mathrm{holds},$$

for a regularization parameter λ to enforce the sparsity of *S*. The more common way to write RNMF follows by using the constraint to replace *N* by X−WH−S (which we do below).

During our initial experiments with Equation (2), we observed that the natural shrinking bias of the $\ell^{1}$ norm (see [47]) significantly harms the performance of the RNMF model, even for small values of λ. On the other hand, λ=0, leads to highly non-unique minimizers of Equation (2) with the undesired trivial solution S=X, WH=0 being one of them.

Therefore, we exploit the idea of Bregman iterations [41] to iteratively replace the (constrained) regularization term $\delta_{\geq 0}\left(S\right)+{∥S∥}_{1}$ by $\delta_{\geq 0}\left(S\right)+{∥S∥}_{1}-\langle p_{S}^{k},S\rangle$ for a subgradient $p_{S}^{k}\in \partial (\delta_{\geq 0}{+∥\cdot ∥}_{1})\left(S^{k}\right)$, where $\delta_{\geq 0}$ denotes the characteristic function of the non-negativity constraint, $S^{k}$ refers to a previous estimate of the mitral valve *S*, and the operation 〈·,·〉 denotes the dot product. More precisely, we alternate between updating WH, *S* and the subgradient $p_{S}$ via the following:

(3) $$\begin{array}{cc} (W^{k+1},H^{k+1})= & \underset{W,H\geq 0}{arg min}{∥X-WH-S^{k}∥}_{2}^{2} \end{array}$$

(4) $$\begin{array}{c} \begin{array}{cc} S^{k+1}= & \underset{S\geq 0}{arg min}{∥X-W^{k+1}H^{k+1}-S∥}_{2}^{2}\\ \; & +\lambda \left({∥S∥}_{1}-\langle p_{S}^{k},S\rangle \right). \end{array} \end{array}$$

(5) $$\begin{array}{cc} p_{S}^{k+1}= & p_{S}^{k}+\frac{1}{\lambda }\left(X-W^{k+1}H^{k+1}-S^{k+1}\right). \end{array}$$

Note that if some component ${\left(S^{k}\right)}_{i,j}$ is positive, it follows that ${\left(p_{S}^{k}\right)}_{i,j}=1$ such that the next regularization term ${∥S∥}_{1}-\langle p_{S}^{k},S\rangle$ no longer penalizes positive values of ${\left(S^{k}\right)}_{i,j}$ at all and therefore naturally removes the shrinking bias. We refer to [47] for details.

The alternations in Equations (3)–(5) are realized by the update steps shown in Algorithm 1, with σ being the thresholded pointwise proximal operator of the $\ell^{1}$ norm:

(6) $$\sigma {\left(u\right)}_{\lambda }=\left\{\begin{array}{cc} u-\lambda & \mathrm{if}\;u>\lambda \\ 0 & \mathrm{otherwise} \end{array}\right.$$

**Algorithm 1:** Update step of W, H, S and p for muscle detection.1:$S_{i,j}\leftarrow \sigma {\left({\left(X-W\cdot H+\lambda p\right)}_{i,j}\right)}_{\lambda }$2:$p_{i,j}\leftarrow p_{i,j}+\frac{1}{\lambda }\left({\left(X-W\cdot H\right)}_{i,j}-S_{i,j}\right)$3:$W_{i,j}\leftarrow W_{i,j}\left(\frac{|{\left(\left(S-X\right)H^{T}\right)}_{i,j}|-{\left(\left(S-X\right)H^{T}\right)}_{i,j}}{{\left(2WHH^{T}\right)}_{i,j}}\right)$4:$H_{i,j}\leftarrow H_{i,j}\left(\frac{|{\left(W^{T}\left(S-X\right)\right)}_{i,j}|-{\left(W^{T}\left(S-X\right)\right)}_{i,j}}{2{\left(W^{T}WH\right)}_{i,j}}\right)$

The optimization steps are followed by a normalization step to acquire a unique solution. For more details, we refer to [26].

Opposed to [41], where Bregman iterations were considered in the context of convex energy minimization problems, the RNMF objective is non-convex such that one cannot expect to compute global minimizers (also not in update Equation (3)), which is why the initialization is important. We initialize S=0 such that the first update of *W* and *H* solves a usual non-negative matrix factorization problem for which we use the multiplicative update algorithm [32].

We iterate Equations (3)–(5) for 100 iterations, with subproblem Equation (3) being approximated by a single step of the multiplicative algorithm. This way, we exploit early stopping before the method converges to a $\ell^{1}$-minimizing solution. We refer the reader to [41] (Theorem 3.5) for more properties of stopping the Bregman iteration early in the convex case and to [48] for more details on the analysis of Bregman iterations in the non-convex case.

### 3.2. Valve Segmentation

This section describes our method to compute the segmentation of the mitral valve as a binary mask $\tilde{B}\in {\left\{0,1\right\}}^{s\times t}$ that is supposed to cover the mitral valve in *X*, i.e., the individual pixel values should behave as follows:

(7) $${\tilde{B}}_{i,j}=\left\{\begin{array}{cc} 1 & \mathrm{if}\;\mathrm{the}\;\mathrm{mitral}\;\mathrm{valve}\;\mathrm{appears}\;\mathrm{in}\;X_{i,j}\\ 0 & \mathrm{otherwise} \end{array}\right.$$

Our segmentation model:

(8) $$\begin{array}{cc} \hat{B}=\underset{B,B_{ij}\in \left[0,1\right]}{arg min} & \frac{1}{2}\langle \Theta -\hat{S},B\rangle +\lambda_{1}TV\left(B\right)+\lambda_{2}\langle 1,B\rangle \\ & +\lambda_{3}\langle \hat{W}\hat{H},B\rangle , \end{array}$$

is based on the segmentation method by Chan and Vese [12] and contains modifications to adapt the problem formulation to our problem of mitral valve segmentation. Instead of minimizing over $\tilde{B}$, we use convex relaxation and minimize over all $B\in {\left[0,1\right]}^{s\times t}$ to convexify our problem.

Here, $\hat{W}$, $\hat{H}$ and $\hat{S}$ are the results of the method in Section 3.1 and are kept fixed. The parameter $\Theta \in R_{>0}$ is a threshold. Since the pixel values $B_{ij}$ of *B* are enforced to grow, if ${\hat{S}}_{ij}>\Theta$, a small value of Θ=0.01 ensures that barely recognizable noise in $\hat{S}$ does not cause the corresponding pixel values in *B* to increase. As a result, this noise is not segmented in *B*. The first regularization term applies (isotropic ) Total Variation (TV) [49] on *B* for a regularization parameter $\lambda_{1}$ with the effect of penalizing the edge length of the segmented area in *B*. In the discrete setting, isotropic Total Variation (TV) is defined as $TV\left(B\right)=\sum_{i=1}^{s}\sqrt{{\left({\left(D_{x}b\right)}_{i}\right)}^{2}+{\left({\left(D_{y}b\right)}_{i}\right)}^{2}}$ (see [50]), where $D_{x}$ and $D_{y}$ are matrices that calculate the gradient on *b* by discretized forward differences in the spatial x- and y directions, and *b* the vectorized form of *B*.

The number of large pixels in *B* is reduced by the second regularization term, which is weighted with $\lambda_{2}>0$ with the intention to remove noise from B. The third regularization term $\langle \hat{W}\hat{H},B\rangle$ penalizes large values in *B*, if there is correspondingly a large pixel value in $\left(\hat{W}\hat{H}\right)$ at the same location. Since $\left(\hat{W}\hat{H}\right)$ contains the rigid heart muscle, this means that an occurrence of muscle structure forces the values in *B* to be small since they are not supposed to be segmented. A reformulation of (8) is given in (9) and leads to another interpretation.

(9) $$\begin{array}{cc} \hat{B}=\underset{B,B_{ij}\in \left[0,1\right]}{arg min} & \frac{1}{2}\langle \Theta_{2}-\hat{S},B\rangle +\lambda_{1}TV\left(B\right)+\lambda_{2}\langle 𝟙,B\rangle ,\\ & \mathrm{with}\;\Theta_{2}=\Theta +2\lambda_{3}\hat{W}\hat{H} \end{array}$$

The threshold values of $\Theta_{2}\in R_{>0}^{s\times t}$ now depend on the entries in $\left(\hat{W}\hat{H}\right)$, as large values of $\left(\hat{W}\hat{H}\right)$ lead to large entries in the threshold $\Theta_{2}$. Thus, the values in *B* at positions with large values in $\Theta_{2}$ only increase if accordingly large pixel values are present at the respective positions in *S*. Therefore, in this formulation, the threshold provides the information at which point the mitral valve occurs with which likelihood, while the third regularization term from Equation (8) is omitted.

The pixel values in *B* are typically close to 0 and 1 after minimizing the energy in (8) or (9). So, to finally obtain a binary segmentation mask $\tilde{B}$, we binarize $\hat{B}$ by thresholding its pixel values at 0.5. To solve (9), we calculate the update steps via the primal–dual hybrid gradient (PDHG) algorithm by Chambolle and Pock [51] by solving the following:

(10) $$\min_{b,b_{i}\in \left[0,1\right]}\max_{p}\langle Db,p\rangle +G\left(b\right)-F^{*}\left(p\right),$$

the primal dual form of (9), with the primal variable *b*, the dual variable *p* and the following:

(11) $$\begin{array}{cc} \; & G\left(b\right)=\frac{1}{2}\langle \Theta_{2,vec}-s,b\rangle +\lambda_{2}\langle 𝟙,b\rangle ,\\ \; & F\left(Db\right)=\lambda_{1}TV\left(b\right). \end{array}$$

The lowercase variables $s\in R^{st}$ and $b\in {\left[0,1\right]}^{st}$ represent the vectorized form of their corresponding uppercase matrices as well as $\Theta_{2,vec}=vec\left(\Theta_{2}\right)$. $F^{*}$ is the convex conjugate form of *F*, and *D* represents the discrete derivative matrix. The update steps of (9) are realized as shown in Algorithm 2. Here $\iota_{\left|\right|\cdot {\left|\right|}_{2,\infty }\leq 1}$ is the convex conjugate of the $\ell^{2,1}$-norm:
**Algorithm 2:** Update step of B for segmentation.1:$p^{k+1}\leftarrow \iota_{\left|\right|\cdot {\left|\right|}_{2,\infty }\leq \lambda_{1}}\left(p^{k}+\sigma D{\bar{b}}^{k}\right)$2:$b^{k+1}\leftarrow b^{k}-\tau \left(D^{T}p^{k+1}+\lambda_{1}\left(t-S\right)+\lambda_{3}WH+\lambda_{2}\right)$3:${\bar{b}}^{k+1}\leftarrow 2b^{k+1}-b^{k}$

### 3.3. Refinement

Due to a strong movement of the heart muscle, it is possible that certain structures that do not belong to the mitral valve are also segmented. In this phase, we refine the segmentation result $\tilde{B}$ in two steps to extract the part of the segmentation mask that only covers the mitral valve.

#### 3.3.1. Calculation of the Centroid

To detect the approximate location of the mitral valve, we consider the spatial centroid of the segmentation video with additional iterative steps alleviating the existence of outliers as shown in Algorithm 3. In the following, ${\hat{S}}_{3D}\in R^{s_{x}\times s_{y}\times t}$ is the result of the previous method, introduced in Section 3.1.

**Figure 2:**
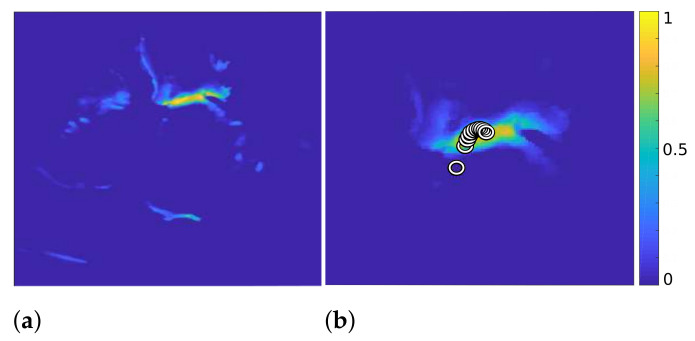
${\tilde{S}}^{1}$ of Algorithm 3 (**a**) and ${\tilde{S}}^{11}$ with the calculated centroids displayed by circles, after 11 iterations ((**b**), zoomed in).

At first, we combine the frames of video ${\hat{S}}_{3D}$ and obtain a 2D map ${\tilde{S}}^{1}\in R_{+}^{s_{x}\times s_{y}}$ with high values in the places where bright structures in ${\hat{S}}_{3D}$ appear most frequently (see Figure 2, left). In the first iteration of our algorithm, we calculate the centroid $C^{1}=\left(C_{x}^{1},C_{y}^{1}\right)$ of ${\tilde{S}}^{1}$ and consider it the position where the mitral valve is most likely to be located. To reduce the influence of the outlier structures on the calculation of the mitral valve location, we multiply ${\tilde{S}}^{1}$ pointwise with a 2D Gaussian function $g(\sigma ,C_{x},C_{y})$ with

$$g_{x,y}\left(\sigma ,C_{x},C_{y}\right)=\frac{1}{2\pi \sigma^{2}}\exp\left(-\frac{{\left(x-C_{x}\right)}^{2}}{\left(2\sigma^{2}\right)}-\frac{{\left(y-C_{y}\right)}^{2})}{\left(2\sigma^{2}\right)}\right),$$

which attenuates structures that are further away from the centroid. Here, $x\in \{1,\cdots ,s_{x}\}$ and $y\in \{1,\cdots ,s_{y}\}$. When the centroid of $S^{2}$ is subsequently recalculated, its position is less influenced by outlier structures.

By iterative continuation of these operations, with decreasing variance σ of the Gaussian function in each iteration, the centroid shifts further toward the mitral valve, as shown on the right in Figure 2. Here, each circle represents the position of the centroid in one iteration. Additionally, the right image in Figure 2 shows the result after eliminating the outlier structures after 11 iterations.
**Algorithm 3:** Iterative calculation of the mitral valve position.1:**for** i =1:11 **do**2:    $v=\sum_{x,y}{\tilde{S}}_{xy}^{i}$3:    $C_{x}^{i}\leftarrow \frac{1}{v}\sum_{x,y}{\tilde{S}}_{xy}^{i}\cdot x$4:    $C_{y}^{i}\leftarrow \frac{1}{v}\sum_{x,y}{\tilde{S}}_{xy}^{i}\cdot y$5:    ${\tilde{S}}^{i+1}\leftarrow {\tilde{S}}^{i}\circ g\left(\sigma ,C_{x}^{i},C_{y}^{i}\right)$6:**end for**7:$C_{fin}=C^{11}$

#### 3.3.2. Clustering

**Figure 3:**
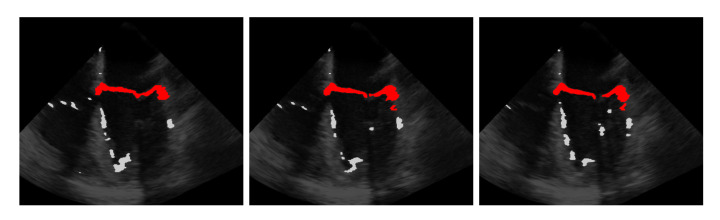
Three frames showing the extraction of the mitral valve (red) from the mis-segmented structures (light gray) by the refinement step.

**Figure 4:**
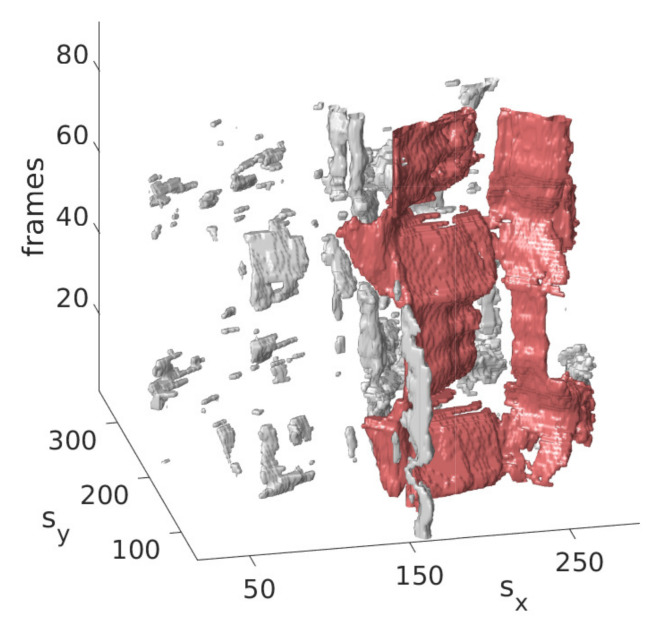
Three-dimensional view of the mitral valve (red), detected by the refinement step.

The idea is that with the information obtained from the last step on the approximate position of the mitral valve, only those segmentations should be retained that are considered likely to cover the mitral valve. For this purpose, the segmentation in $\tilde{B}$ is interpreted as a set of connected components, in the following referred to as clusters $\left\{U_{i}\right\}$ with each $U_{i}=\left\{u_{1}^{i},\cdots ,u_{n^{i}}^{i}\right\}$ being a set of connected pixels, where pixels of the binary video $\tilde{B}$ that are equal 1 and connected in temporal and spatial dimensions form a cluster as shown in Figure 3 exemplarily in three successive frames and in Figure 4 in a three-dimensional visualization of the detected mitral valve.

In a purely spatial clustering, the alternately closing and opening valve leaflets do not touch each other and thus form two clusters in the open state, whereas in the closed state, only one cluster is formed due to the contacting valve leaflets. Because of this, it is particularly important to form the clusters not only in the spatial dimension, but also in the temporal dimension to ensure that only a single cluster covers the mitral valve. To find the cluster $U_{\hat{i}}$ which is closest to the centroid $C_{fin}$, we measure the smallest distance of each cluster $U_{i}$ to $C_{fin}$ by first finding in each cluster the pixel $u_{{\hat{p}}^{i}}^{i}$ that is closest to the centroid with

(12) $${\hat{p}}^{i}=\underset{p}{arg min}{∥u_{p}^{i}-C_{fin}∥}^{2},$$

measuring the distance and selecting the cluster $U_{\hat{i}}$ with the smallest distance with the following:

(13) $$\hat{i}=\underset{i}{arg min}{∥u_{{\hat{p}}^{i}}^{i}-C_{fin}∥}^{2}.$$

### 3.4. Windowing

A given mitral valve size can be used to restrict the segmentation of the mitral valve to a localized area of interest. To detect this area of interest, we first solve our model as follows:

(14) $$\underset{W,H,S\geq 0}{arg min}{∥X-WH-S∥}_{2}^{2}+\mu_{1}{\left||S|\right|}_{1}+\mu_{2}\langle WH,S\rangle$$

on the input echocardiographic video *X*; Equation (14) is similar to Equation (2), but with an additional regularization to enforce exclusionary entries in (WH) and *S* for a regularization parameter $\mu_{2}>0$. A large value in (WH) is accompanied by a small value in *S* and vice versa. Then, we move a window $W\in R^{s_{x}^{W}\times s_{y}^{W}\times t}$ with $s_{x}^{W}<s_{x}$ and $s_{y}^{W}<s_{y}$ along the spatial dimensions of the minimizer $\hat{S}\in R^{s_{x}\times s_{y}\times t}$ of Equation (14). For every window position, we calculate the squared Frobenius norm $c:=\left|\right|\hat{S}{\left|\right|}_{F}^{2}$ of the values in $\hat{S}$ that are covered by the window. The position of the window with the maximal resulting *c* is most likely to cover the mitral valve. Unlike [9], we do not solve Equation (14) on every window position, but only once on the whole video. This is computationally less expensive and in our case, provides more precise window positions covering the mitral valve. To solve the minimization problem Equation (14), we alternate between updating *W*, *H*, *S* and the subgradient $p_{s}$ in an iterative way. The update steps of *W*, *H* and *S* are similar to Equations (3) and (4), but this time, under the consideration of the new regularization term:

(15) $$\begin{array}{c} \begin{array}{cc} (W^{k+1},H^{k+1})= & \underset{W,H\geq 0}{arg min}{∥X-WH-S^{k}∥}_{2}^{2}\\ \; & +\mu_{2}\langle WH,S^{k}\rangle , \end{array} \end{array}$$

(16) $$\begin{array}{c} \begin{array}{cc} S^{k+1}= & \underset{S\geq 0}{arg min}{∥X-W^{k+1}H^{k+1}-S∥}_{2}^{2}\\ \; & +\mu_{1}\left({∥S∥}_{1}-\langle p_{S}^{k},S\rangle \right)\\ \; & +\mu_{2}\langle W^{k+1}H^{k+1},S\rangle . \end{array} \end{array}$$

Similar to Algorithm 1, the updates are realized as shown in Algorithm 4, σ being the proximal operator of the $\ell^{1}$ norm with the threshold (see Equation (6)):
**Algorithm 4:** Update step of W, H, S and p for windowing.1:$S_{i,j}\leftarrow \sigma {\left({\left(X-WH+\mu_{1}p-\mu_{2}WH\right)}_{i,j}\right)}_{\mu_{1}}$2:$p_{i,j}\leftarrow p_{i,j}+\frac{1}{\mu_{1}}\left({\left(X-WH\right)}_{i,j}-S_{i,j}-\mu_{2}{\left(WH\right)}_{i,j}\right)$3:$W_{i,j}\leftarrow W_{i,j}\left(\frac{|{\left(\left(S-X+\frac{1}{2}\mu_{2}S\right)H^{T}\right)}_{i,j}|-{\left(\left(S-X+\frac{1}{2}\mu_{2}S\right)H^{T}\right)}_{i,j}}{{\left(2WHH^{T}\right)}_{i,j}}\right)$4:$H_{i,j}\leftarrow H_{i,j}\left(\frac{|{\left(W^{T}\left(S-X+\frac{1}{2}\mu_{2}S\right)\right)}_{i,j}|-{\left(W^{T}\left(S-X+\frac{1}{2}\mu_{2}S\right)\right)}_{i,j}}{2{\left(W^{T}WH\right)}_{i,j}}\right)$

## 4. Dataset

**Figure 5:**
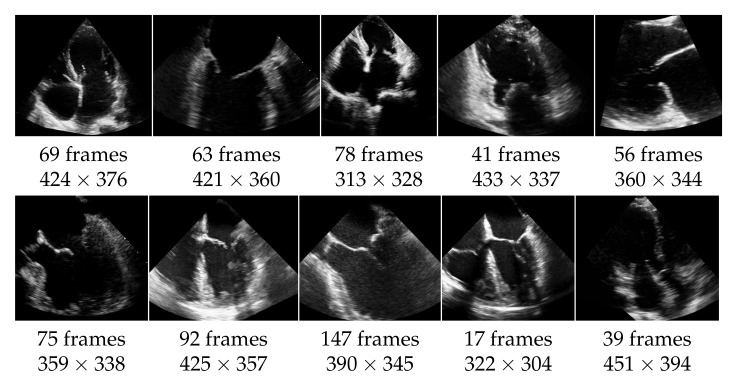
Dataset of 10 echocardiographic videos, with their number of frames and their spacial resolution.

The echocardiographic videos used for the evaluation were obtained from the Keck Medical Center of the University of Southern California. This study was approved by the institutional review board at the University of Southern California (Protocol ID: HS-15-00258). The dataset contains 10 different recordings of the heart in multiple views as shown in Figure 5. The analysis of the mitral valve segmentation was performed from these views, and the sample included the most common types of mitral valve pathology in addition to normal valve anatomy. Three cases show an abnormality of the mitral valve, specifically of the posterior mitral valve leaflet called flail mitral valve. This is a variation of a mitral valve prolapse category. The other videos do not show clear evidence of mitral valve pathology. The ultrasound videos differ also in length (from 17 to 147 frames) and in quality, with side lengths from 304 to 451 pixels, and have a frame rate of 30 frames per second. The mitral valve appears differently pronounced in the videos, which causes different results also regarding the detection as well as the segmentation. For each video frame, the ground truth of the segmentation consists of a contour that encloses the edges of the mitral valve and was manually created by non-medical experts and approved by an echocardiographer expert.

In addition, we evaluate our method on 46 videos from the EchoNet Dynamic dataset (see [52]), selected in [40]. The videos have a resolution of 112×112 pixels, which is lower than the resolution of our previously introduced dataset. For evaluation, we use the ground truth labeling provided by [40].

For the results on this dataset, we refer to Section 5.5.

## 5. Results

In the following, we compare our method with [9,40] according to the f1-score. We distinguish between the automatic method, in which no windowing is applied, and the method in which additionally a window slides over the video. Furthermore, we consider the recall regarding the windowing method, which is the number of ground truth pixels that are covered by the window with respect to the whole number of ground truth pixels. This gives us a measure of how much of the mitral valve is covered by the window. The hyperparameters used for the experiments are given in Table 2 for the muscle detection and valve segmentation algorithms and in Table 3 for the window detection algorithm. The hyperparameters for [40] are set as indicated in [40]. In agreement with the authors, we use for the experiments with [9] rank 2, a sparseness coefficient of 0.2, and for the threshold function, the top 1% pixels in terms of intensity.

### 5.1. On Bregman Iteration and RNMF

**Figure 6:**
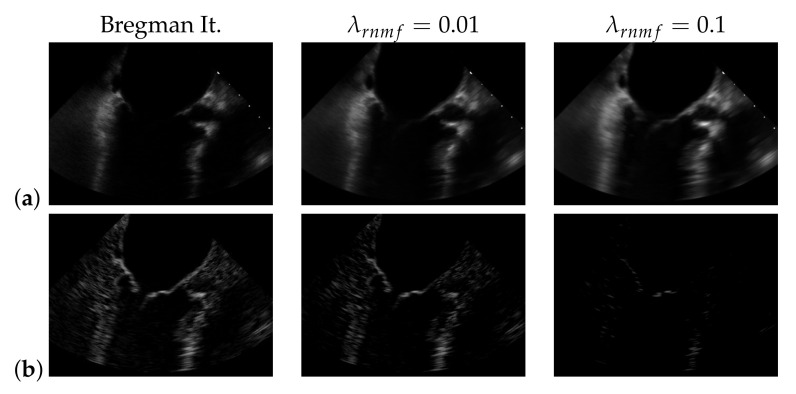
$\left(\hat{W}\hat{H}\right)$ (**a**) and $\hat{S}$ (**b**) in case of using Bregman iteration and of standard robust non-negative matrix factorization (RNMF) for different values of the regularization parameter $\lambda_{rnmf}$.

**Figure 7:**
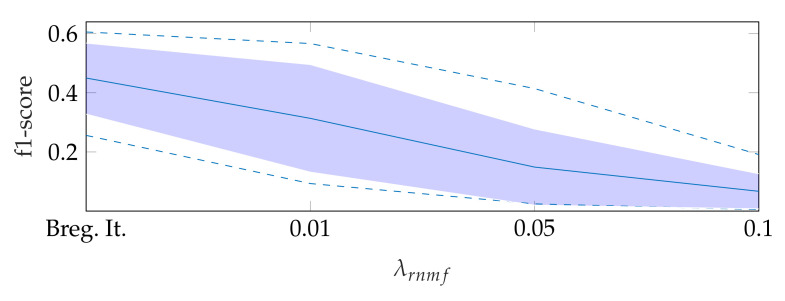
F1-score for automatic segmentation depending on $\lambda_{rnmf}$, where the dashed lines show the minimum and maximum f1-score values, and the standard deviation is indicated by the filled range.

To find the heart muscle structures, we use RNMF with Bregman iteration (see Section 3.1). For the subsequent segmentation, it is of great importance that no heart valve structures are visible in $\left(\hat{W}\hat{H}\right)$ because structures that appear in $\left(\hat{W}\hat{H}\right)$ are penalized, so they do not appear in the segmentation result. In the following, $\lambda_{rnmf}$ refers to the hyperparameter λ of RNMF without Bregman iteration. As shown in Figure 6, increasing $\lambda_{rnmf}$ pushes parts of the mitral valve into the product $\left(\hat{W}\hat{H}\right)$, which our segmentation algorithm assumes not to contain the mitral valve. In addition, we observe in videos with a low contrast mitral valve that the mitral valve is partly removed from the result $\hat{S}$ with increasing $\lambda_{rnmf}$. The f1-score for *automatic segmentation* decreases when increasing $\lambda_{rnmf}$ as shown in Figure 7: with Bregman iteration, the f1-score is 0.45; for $\lambda_{rnmf}=0.01$, the f1-score is 0.314; and for $\lambda_{rnmf}=0.1$, the f1-score is 0.066.

### 5.2. Automatic Segmentation

**Figure 8:**
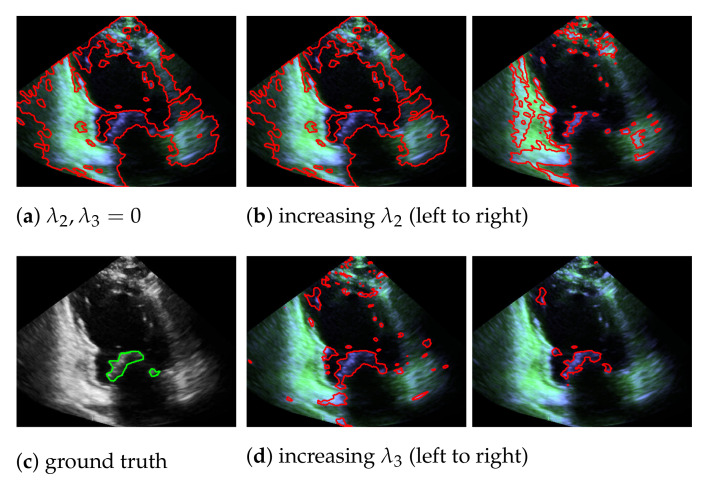
Segmentation of the mitral valve (red contour), depending on $\lambda_{2}$ and $\lambda_{3}$ of Equation (8), with the entries in $\hat{W}\hat{H}$ appear in green and the entries in $\hat{S}$ in blue; the ground truth is highlighted with a green contour.

In our mitral valve segmentation method that does not include the windowing step (see Section 3.4), we first split the muscle and the mitral valve structure as described in Section 3.1 by solving Equation (2), followed by the segmentation step of Section 3.2. Here, a strong weighting of the third regularization term in Equation (8) has a positive effect on the segmentation of the valve, as structures that are not located in the area of the muscle structure are preferably segmented. This is shown in Figure 8, by showing the behavior of the segmentation depending on $\lambda_{2}$ and $\lambda_{3}$, whereby a large value in $\lambda_{3}$ provides better capturing of the mitral valve than an increase of $\lambda_{2}$.

**Figure 9:**
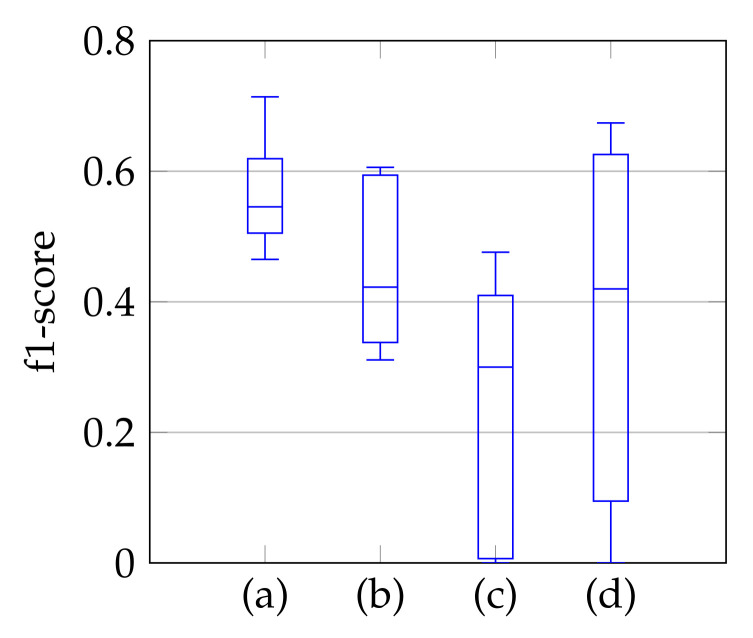
F1-score of segmentation result over ten videos for segmentation with windowing (**a**), automatic segmentation (**b**), segmentation by Dukler et al. (**c**) and segmentation by Corinzia et al. (**d**).

As a third step, we remove the segmented noise with the refinement that is described in Section 3.3. The resulting f1-scores compared to the ground truth are visualized in Figure 9 in the form of boxplot diagrams for segmentation, using the *windowing* approach in (a), for the method of Dukler et al. [9] in (c) and for the method of Corinzia et al. [40] in (d). The f1-score for *automatic segmentation* is shown in (b) and shows a slightly lower median f1-score than the one in (a), but without conspicuous negative outliers regarding the segmentation accuracy. Outliers indicate a complete missing of the mitral valve, which is not the case here. In [40], there are shown outliers, whereas [9] has a slightly lower f1-score with a larger variation. In addition to the boxplot diagrams, the average recall, precision, and f1-score values are given in Table 4.

**Figure 10:**
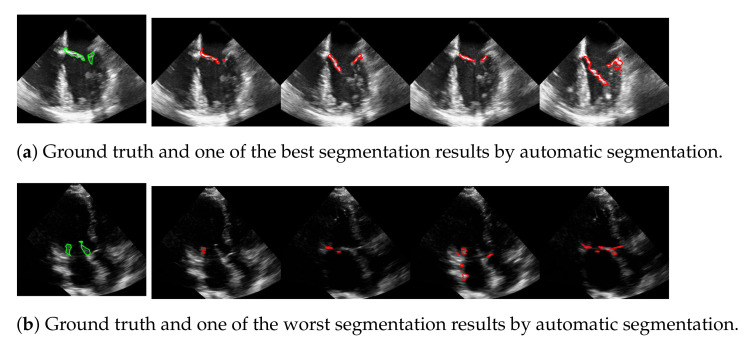
One of the best (**a**) and the worst (**b**) segmentation results by automatic segmentation (red), with the corresponding ground truth (green).

The advantage of this method is that the mitral valve segmentation is performed without specifying a window size. In order to obtain a visual impression of the results, one of the best and the worst segmentations with respect to the f1-score are shown in Figure 10. Here, the example in the last row is particularly challenging because the mitral valve moves very quickly, as well as the heart muscle, and partially disappears in some frames. Nevertheless, the correct position of the valve is detected.

**Figure 11:**
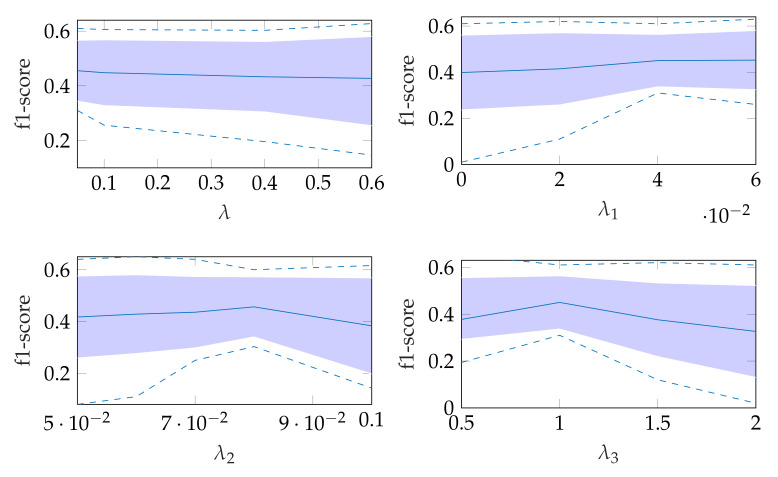
Effect of changing segmentation parameter for automatic segmentation, where the dashed lines show the minimum and maximum f1-score values and the standard deviation is indicated by the filled range.

The robustness of the automatic segmentation method against variations in the hyperparameters is shown in Figure 11, which shows the changes in the f1-score when changing the hyperparameter values in Equation (8). In each of these graphs, one parameter was varied and the others were fixed as specified in Table 2.

### 5.3. Windowing

**Figure 12:**
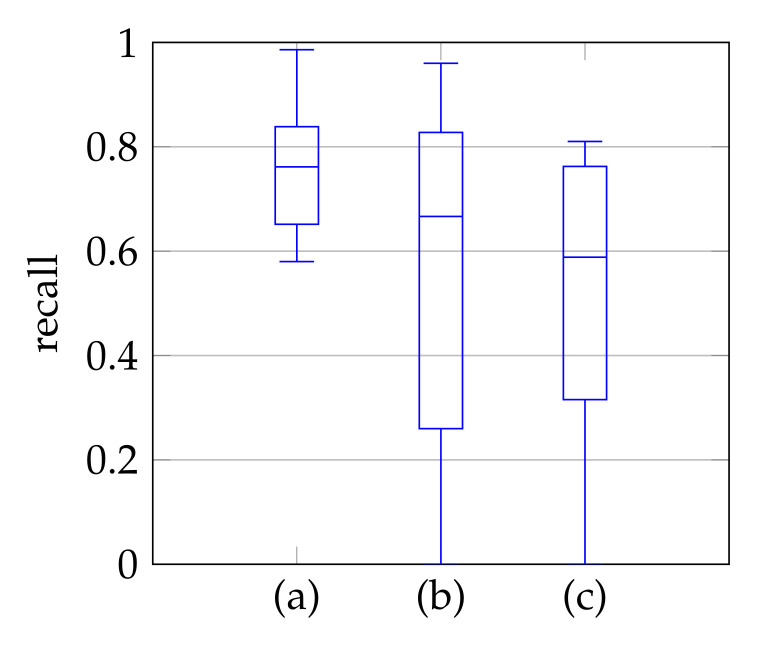
Recall of mitral valve detection over ten videos for Windowing (**a**), detection by Dukler et al. (**b**) and detection by Corinzia et al. (**c**).

**Figure 13:**
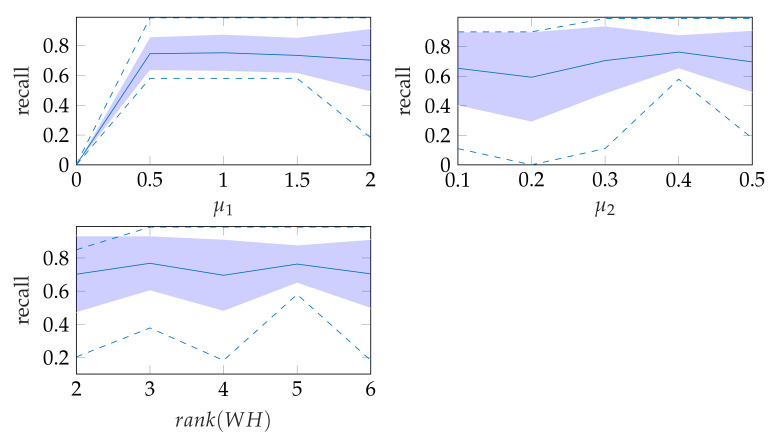
Robustness of windowing parameters, where the dashed lines show the minimum and maximum recall values and the standard deviation is indicated by the filled range.

In window search, it is important that as much of the mitral valve structure as possible is covered by the window. Different views of the heart are accompanied by different sizes of the mitral valve appearance in the echocardiographic video. Therefore, it is necessary to know the required size of the window to cover the valve in the respective video if many different views of the heart should be supported by the method. To determine the amount of mitral valve structure that is covered by the window, we measure the recall of the ground truth and the detect window position (see Section 3.4). These values are compared to the recall values obtained by applying the method of Dukler et al. [9] and the method of Corinzia et al. [40] and visualized in the form of boxplot diagrams in Figure 12. We see that our method detects the mitral valve in the median more accurately and without negative outliers. Again, these outliers with a recall near zero indicate a missed mitral valve. The robustness of the *windowing* method against variations in the hyperparameters is shown in Figure 13, which show the changes in the mean recall when changing the hyperparameter values of the method. In each of these graphs, one parameter was varied and the others were fixed as specified in Table 3.

### 5.4. Segmentation with Windowing

**Figure 14:**
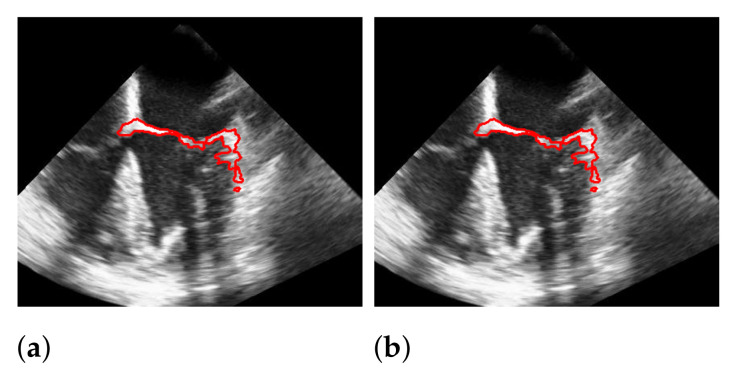
Effect of applying windowing on segmentation. The red marks indicate the segmentation. (**a**) without windowing; (**b**) with windowing.

To segment the mitral valve, we pass the steps of Section 3.1–Section 3.3 with almost the same parameter settings as the method that does not include the windowing step and limit additionally the area in which the mitral valve may appear as described in Section 3.4. The effect of windowing is illustrated in Figure 14, which shows a segmentation with and without windowing. Without windowing, structures that are close to the mitral valve and move strongly may be segmented. These cases are handled by additional windowing. Since the windowing step limits the spatial range of the segmentation, here the weight $\lambda_{2}$ in (8) is set to a slightly lower value, compared to the segmentation without the windowing step, with the effect that more structure is detected as the mitral valve structure, and thus, the chance of potentially not capturing the whole valve is reduced.

**Figure 15:**
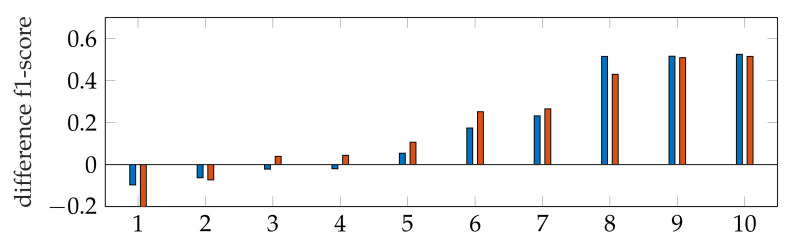
Ascending ordered difference of the f1-score for 10 videos: f1-score of segmentation with windowing subtracted by the f1-score of Dukler et al. (blue) and f1-score of segmentation with windowing subtracted by the f1-score of Corinzia et al. (red).

The f1-score of the segmentation is in general larger with applying *windowing* instead of *automatic segmentation*, as shown in Figure 9. In addition, our segmentation achieves better results than [9,40]. By subtracting the resulting f1-scores of Dukler et al. [9] and Corinzia et al. [40] from the f1-scores of *segmentation with windowing* for each video, we obtain a comparison of the accuracy of these two methods for multiple echocardiographic videos as shown in Figure 15.

Here, the blue bars correspond to the measured differences with [9], and the red bars correspond to the differences with [40]. For both methods, it can be seen that the segmentation accuracy deviates less from ours in cases where the methods [9,40] are better (left bars) than in cases where our method has better accuracy (right bars).

**Figure 16:**
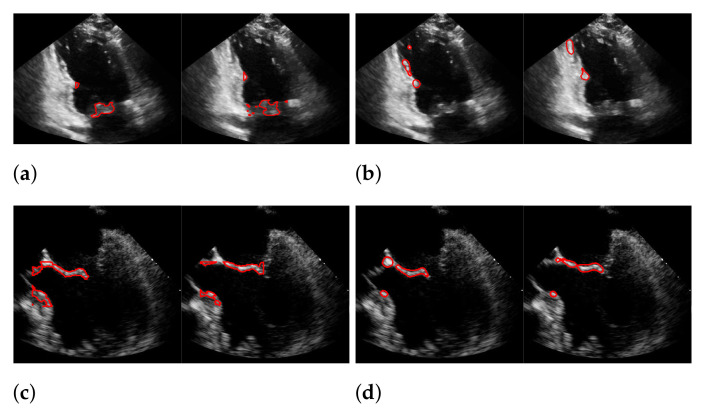
Segmentation in the cases of the largest differences between the f1-scores of our approach (segmentation with windowing) (**a**,**c**) and the one of Dukler et al. (**b**,**d**). The results in (**a**,**b**) belong to the blue rightmost bar of Figure 15, the results in (**c**,**d**) belong to the leftmost bar.

**Figure 17:**
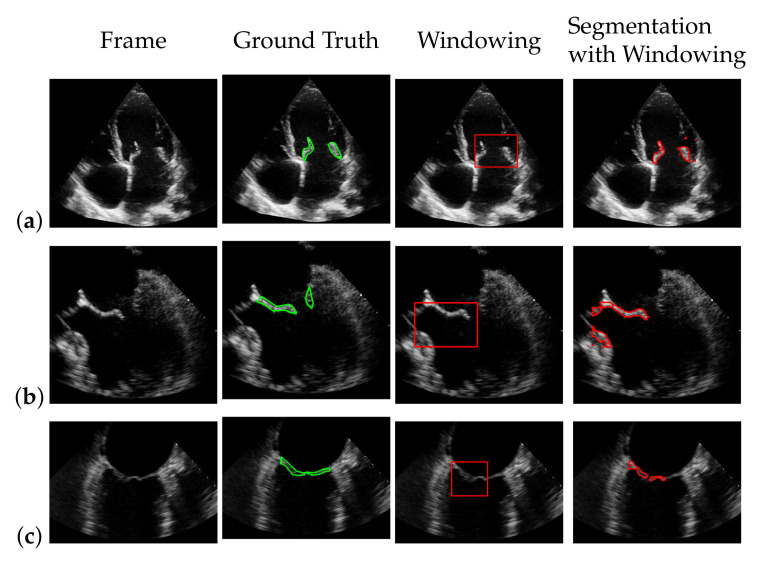
Best (**a**), worst (**b**) and an average (**c**) detection and segmentation result in terms of the recall and f1-score. The green contour indicates the ground truth segmentation, the red contour indicates the detected window location and the calculated segmentation.

**Figure 18:**
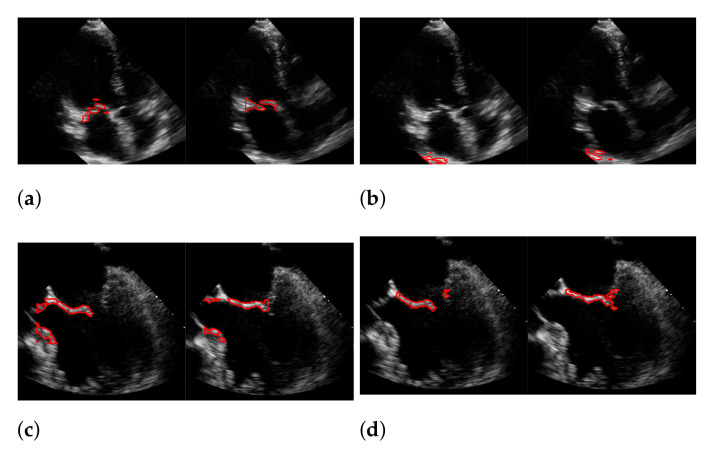
Segmentation in the cases of the largest differences between the f1-scores of our approach (segmentation with windowing) (**a**,**c**) and the one of Corinzia et al. (**b**,**d**). The results in (**a**,**b**) belong to the red rightmost bar of Figure 15, the results in (**c**,**d**) belong to the leftmost bar.

For Dukler et al. [9], the segmentation results that correspond to the leftmost and the rightmost bars in Figure 15 are shown in Figure 16. The corresponding ground truths of the videos are shown in Figure 8 and Figure 17. For Corinzia et al. [40], the segmentation results that correspond to the leftmost and the rightmost bars in Figure 15 are shown in Figure 18.

In both figures (Figure 16 and Figure 18), the upper row of the segmentation by [9,40] gives an example of a missed mitral valve using a windowing technique and underlines the importance of correct valve detection. The upper row videos are especially challenging because of a partly disappearing mitral valve and weak contrast.

The bottom rows show the result on a video, including the mitral valve and the aortic valve on the left, which is also segmented. Since the final segmentation accuracy strongly depends on the windowing result the best/worst f1-score of the *segmentation with windowing* and the best/worst recall value of the *windowing* method on our evaluation dataset refer to the same videos as expected. These are shown in Figure 17. Here, the first row shows the best result and the second row, the worst. The latter is, again, caused by the second valve on left, which is also moving fast. The last row shows an average result of the mitral valve detection and segmentation.

**Figure 19:**
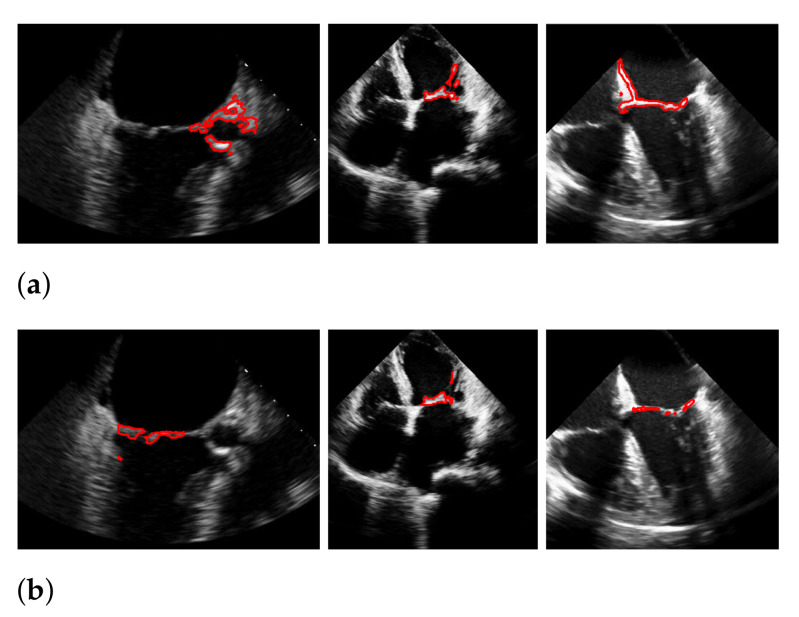
Types of segmentation failures. (**a**) segmentation of Corinzia et al.; (**b**) segmentation with windowing.

During our experiments, we observed different kinds of failures in the segmentation with windowing, which we illustrated in Figure 19 with three example frames. The most far-reaching failure, which appears in the form of a very low f1-score, is the complete missing of the mitral valve during windowing, as shown in the upper left image, and can occur in methods with windowing, as here in the method of Corinzia et al. [40]. The two images in the middle show failures that can occur due to the rectangular shape of the windows, where in some frames, parts of the heart muscle are segmented, as it is located in the area of the window during the contraction of the heart. The third type of failure is a slightly shifted window, as seen in the upper right image, which can occur when one valve leaflet stands out, due to strong pixel intensity and strong surrounding muscle movement in contrast to the other leaflet as well as when a second valve near the mitral valve appears in the video. This failure can also be seen in Figure 17 in the middle image in our worst result. Failures, which can occur in our method, due to too strong regularization of $\lambda_{1}$ or $\lambda_{2}$ in (8), are partially too narrow, perforated segmentations as shown in the lower right image. Here, the valve is correctly localized, but is not completely captured in some frames of the video.

### 5.5. Results on the EchoNet-Dynamic Dataset

For evaluation of the EchoNet-Dynamic dataset [52], we used the same hyperparameters as listed in Table 2 and Table 3 for 46 selected videos, which almost all have the characteristic of showing a second heart valve besides the mitral valve. We measured an f1-score of 0.42 for *segmentation with windowing* and 0.35 for *automatic segmentation*, despite the presence of multiple valves in the videos. On the EchoNet-Dynamic dataset, [40] achieved an f1-score of 0.3 with their proposed method in combination with an optical flow approach. By omitting the optical flow, which is not optimal for the given data, due to its low resolution, they were able to increase the f1-score to 0.53. For the method of [9], we measured an f1-score of 0.1, due to shifts in their windowing approach.

As our method is not designed to distinguish between two valves, we performed additional experiments after masking the second heart valve from the videos and were thus able to increase the segmentation accuracy to an f1-score of 0.51 for *segmentation with windowing* and 0.43 for *automatic segmentation*. At this point, we refer to the conclusion for discussion to increase the robustness of our method against the presence of multiple valves in echocardiographic videos in future work.

## 6. Discussion

In this work, we propose a fully automatic and unsupervised method to detect and segment the mitral valve from echocardiographic videos by using a bias-free version of the RNMF model. In combination with a window-based localization approach and a modified version of the Chan–Vese segmentation method, we could improve the average f1-score value of the resulting segmentation by 0.18, compared to [40] on a dataset with 10 echocardiographic videos to a f1-score of 0.56. Furthermore, a possibility to segment the mitral valve without knowing its spatial size is introduced. The used technique, which employs a newly developed refinement method, is accompanied by a slight loss in accuracy.

During the experiments, we observed that failing cases included mitral valves with low contrast, fast movement of the heart muscle and recordings including more than one heart valve. However, we discovered that even in the video cases where the mitral valve is difficult to detect and segment because it partially disappears or is only in a slight contrast to the background, the correct position of the valve is detected. The method introduced in this paper represents a step toward fully automated mitral valve segmentation. However, especially when segmenting an anatomy as challenging as the mitral valve, it is essential that the quality of the results are critically controlled and not blindly trusted.

Our method offers some advantages, but also has weaknesses that need to be addressed in future work if applied in real medical settings. A limitation of our method is its time consumption since it takes a little over 8 min to calculate the segmentation of an ultrasound video with about 40 frames on an Intel(R) Core(TM) i7-7700HQ CPU @ 2.80 GHz. Therefore, the method is not suitable for time critical scenarios, such as intra-operative guidance, but could be sped up by implementing it on GPU. In addition, the method in [9] is not a real-time method, nor is the method in [40], which requires 13 ± 8 min per video for mitral valve segmentation on a GeForce GTX 1060 GPU but also offers a possibility for time-critical scenarios for which viability was shown in [40]. Furthermore, it should be noted as a weakness that the accuracy of our method, as well as that of the methods of [9,40], is not sufficient to be actually applied in real-life scenarios.

A strength of our method is that we avoid the inherent bias of $\ell^{1}$-regularization in solving the RNMF-model by using Bregman iterations, which gives us the promising advantage of balancing bias avoidance and uniqueness, unlike the method of [9], who uses classical RNMF and unlike the method in [40]. Corinzia et al. [40] compute first the cardiac muscle structures by the neural matrix factorization model and, in a subsequent step, the mitral valve structures by a thresholding network, which predicts a sparse signal, similar to the classical RNMF approach, without avoiding the inherent bias of the $\ell^{1}$-regularization. Another advantage of our method is that we can obtain valid segmentation results that are independent of a windowing method, as we use variational segmentation [12] and a refinement step instead of a simple subtraction of the cardiac muscle structures, as is the case in [9]. Dukler et al. [9] perform RNMF twice to extract the muscle structure from the data. The mitral valve is segmented by subtracting the muscle structure from the original video, which makes a windowing step crucial since no mechanism captures mis-segmented structures. Additionally, [40] need a windowing for their segmentation step since they obtain their resulting segmentation of the mitral valve by morphological opening followed by a connected component approach on the trained sparse signal in the windowed region of interest. Both our method and that of [9] use window detection (which is optional in our case); however, our method includes additional regularization that has the benefit of eliminating the co-occurrence of the muscle and valve on the same pixel. Both of these methods have the disadvantage that they cannot distinguish between the two heart valves. A solution to this problem is introduced in [40] in form of a time-varying weighting during windowing. The weighting depends on the phase of the cardiac cycle and thus allows to distinguish between right and left heart valves since the valves open and close at different time intervals.

In the future, it would be interesting to combine our methods with those in [40] to increase robustness. In particular, the time-variant weighting presented in [40] improves the detection accuracy of the mitral valve for cases where more than one valve is shown in the echocardiographic video. Since our windowing method has no mechanism to distinguish between the two valves, the introduction of time-varying weighting would likely increase segmentation accuracy. Additionally, for the fully automated method, time-variant weighting could be useful as a certainty measurement to distinguish low certain segmentation from high certain segmentation, and to improve the first mentioned by, for example, interpolating the high certain segmentation. This would be of particular interest for fully removing the windowing step from the mitral valve segmentation pipeline to avoid failures and remove the dependency on window sizes.

## Figures and Tables

**Table 1:** Summary of mitral valve segmentation methods for echocardiographic recordings.

Method	Category	Prior Knowledge	Pros and Cons	Paper
Energy Minimization Method	Active Contours	Initial Contour	Reduces the effort of the manual segmentation of each frame to the segmentation of only one frame. However, the disadvantage is that there has to be prior knowledge of the position of the mitral valve and it has to be drawn in by an expert. In addition, a tuning of parameters is necessary.	[8,21,22,23,24]
			While this method has the advantage that no prior knowledge of mitral valve position is required, it has the disadvantage of requiring an extensive parameter adjustment.	[7]
	Matrix Factorization	Mitral Valve Size	These methods do not require a prior drawn contour of the mitral valve in the first frame, but the size of the mitral valve for a windowing method must be known in advance. In addition, an adjustment of parameters is also necessary here.	[9,10]
	Matrix Factorization with Bias Avoiding	Optional: Mitral Valve Size	An advantage of this method is that the size of the mitral valve does not need to be known (optional), but the disadvantage is the required adjustment of parameters.	ours
Machine Learning Method	Unsupervised, Videowise Training	Mitral Valve Size	These methods do not require a prior drawn contour of the mitral valve in the first frame, but the size of the mitral valve for a windowing method must be known in advance. In addition, an adjustment of parameters is also necessary here.	[39,40]
	Supervised	Training Data	An automatic segmentation method without parameter adjustment for each video. However, a drawback is that training data and annotations must be available, which is especially difficult for medical data. In addition, there is an unknown bias shift of segmentation toward the training data.	[13]

**Table 2:** Hyperparameters of the proposed methods automatic segmentation (a), segmentation with windowing (b) for muscle detection and valve segmentation algorithms.

	λ	$\lambda_{1}$	$\lambda_{2}$	$\lambda_{3}$	Rank (WH)
(a)	0.1	0.04	0.075	1.0	2
(b)	0.1	0.04	0.05	1.0	2

**Table 3:** Hyperparameters of the Windowing method.

$\mu_{1}$	$\mu_{2}$	Rank (WH)
1	0.4	5

**Table 4:** Average recall, precision and f1-score using segmentation with windowing (a), automatic segmentation (b), segmentation by Dukler et al. (c) and segmentation by Corinzia et al. (d).

	Recall	Precision	f1-Score
(a)	0.494	0.692	0.565
(b)	0.44	0.558	0.45
(c)	0.165	0.551	0.244
(d)	0.378	0.43	0.377

## Data Availability

Restrictions apply to the availability of these data. The dataset is provided to the authors by the Keck Medical Center of the University of Southern California.
